# Stirring glycopeptides away from the constraints of solid-phase synthesis misconceptions

**DOI:** 10.3389/fmolb.2026.1863326

**Published:** 2026-06-10

**Authors:** Dror Ben Abba Amiel, Chaim Gilon, Mattan Hurevich

**Affiliations:** 1 Institute of Chemistry, Hebrew University of Jerusalem, Jerusalem, Israel; 2 Center of Nanoscience and Nanotechnology, Hebrew University of Jerusalem, Jerusalem, Israel

**Keywords:** diffusion, glycopeptides, mixing, post-translation modifications, solid-phase synthesis

## Abstract

Glycosylation is one of the most prevalent post-translational modifications of proteins. Synthetic glycopeptides give access to protein fragments with well-defined glycosylation sites, providing a unique route to obtain relevant biochemical information. Since glycosylation can be extremely abundant and appear in different patterns, the assembly of glycopeptide libraries that manifest this variety is required. Several limitations in state-of-the-art solid-phase peptide synthesis make these processes less appropriate for the accelerated preparation of glycopeptides. Our lab has developed a highly-efficient method for glycopeptide synthesis, which employs high-shear mixing at a high temperature to obtain glycopeptides within minutes with minimal waste of building blocks. The development of this new process was not trivial. It encountered synthetic difficulties associated with the complexity of glycan chemistry, which were met by expanding the traditional technological boundaries and challenging common practices. In this perspective, we describe the thought process that has guided us through the development of this method. We illustrate the key role diffusion properties hold for the optimization of reactions and for streamlining and expediting the protocol. We then elaborate on how the ability to question some conceptual bottlenecks associated with SPPS conceptions was pivotal to the success of this project. We compare the presented study with other techniques that aim to accelerate the synthesis of glycopeptides. Finally, we describe the present and future possibilities of the strategy and how they may contribute to expanding the scope of glycopeptide research.

## 1 Introduction

Glycosylation is one of the most prevalent post-translational modifications (PTMs) of proteins (Apweiler et al., 1999). Glycans play a large variety of roles in protein structure, function, and interactions (Shental-Bechor and Levy, 2009; Endo, 2015; Varki, 2017; Pomin and Mulloy, 2018; Potel et al., 2025). Elucidation of glycan influence on proteins is among the major challenges of biochemistry (Hang et al., 2022). Synthetic glycopeptides (GPs) give access to glycoprotein fragments with well-defined glycosylation sites, providing a unique route to obtain relevant biochemical information. Since glycosylation can be extremely abundant and appear in different patterns, the assembly of glycopeptide libraries that manifest this variety is required (Quartararo et al., 2020). However, while peptide libraries are commonly used in research and are manufactured commercially, obtaining GP libraries is limited by the inherent synthetic difficulties (Mehta et al., 2020).

Seminal works that describe the SPPS of O- and N- linked GPs showcased the great potential in the strategy, but also expose some of its limitations (Nakahara et al., 1996; Zhuang et al., 2023). The synthesis of EPO (Wang et al., 2013), Syndecan-1 (Yang et al., 2012), PSGL-1 (Koeller et al., 2000), and mucin (Fumoto et al., 2005) fragments proved that large and complex glycan cores can be installed on peptides via SPPS. The use of convergent and semisynthetic approaches as complementary to the synthetic one provides additional routes to access complex GPs (Ullmann et al., 2012; Wu et al., 2026). The sequential Solid phase synthesis of both the glycan and the peptide in the same process have also been attempted (Paulsen et al., 1997; Hurevich and Seeberger, 2014). To date, the synthesis of a single GP with complex glycans is done usually via multistep, low yielding processes, which makes their procurement extremely challenging (Nagasaki et al., 2016). As a direct outcome of the synthetic limitations, the preparation of GP libraries via standard SPPS strategies is not straightforward.

### 1.1 The synthesis of glycopeptides

The most common method for the synthesis of glycopeptides and of peptides is solid-phase peptide synthesis (SPPS) (Merrifield, 1963; 1986). The solid-phase process utilizes solid-support-anchored active sites, on which all reactions occur. The process consists of several parts, which are in turn comprised of individual steps. Each step is a chemical transformation that includes one or more reactions. The major parts of the process are assembly, in which the peptide backbone is constructed via coupling and deprotection steps, and post-assembly, which includes other modifications of the peptide backbone or cleavage thereof from the solid support.

The synthesis of GPs relies on the use of glycosylated amino acids (GAAs) as pre-synthesized building blocks for SPPS (Seitz, 2000). This approach has many difficulties, starting from the acquisition of protected GAAs, through the assembly of the GP, to the deprotection of the assembled product.

The acquisition of GAAs is hindered by their challenging synthesis. The glycosylation of amino acids requires the use of several orthogonal protecting groups (PGs) and a high level of stereo- and regioselectivity to obtain relevant products (Chen and Xie, 2018). As the preparation of protected GAAs is troublesome, their prices are also orders of magnitude higher than standard protected AAs, making them a precious commodity for SPPS whether they are synthesized or purchased (Frank et al., 2025).

The use of protected GAAs for SPPS often leads to low-yielding coupling reactions, as their bulky protected glycan moieties make them more sterically hindered than regular protected AAs. The common SPPS way to overcome low-yielding coupling reactions is to increase protected AA equivalents and to elongate reaction times. The excessive use of protected GAAs is impractical, considering the difficulty in obtaining them, and the elongation of coupling times increases racemization, thus lowering the yield and making the synthesis of GPs time-consuming.

GAAs are often protected by acetyl groups, necessitating a post-cleavage deacetylation step which is usually done in solution (Behren et al., 2023). The post-cleavage manipulation of peptides is usually highly inefficient, as it requires at least a rudimentary purification prior to the reaction and another purification following it (Behren et al., 2023). Post-cleavage purification steps consume a significant amount of time and lead to loss of yield. Post-cleavage steps also prevent streamlining the process, which makes GP preparation less attractive for the peptide synthesis community.

These challenges make the synthesis of biologically-relevant GPs a slow, expensive, and inefficient process. The aforementioned difficulties are exacerbated when GPs with bulkier glycan moieties or multiple glycosylation sites are synthesized. The traditional SPPS strategy is not streamlined with steps required for GP preparation and has significant consequences in reaction time and in GAA consumption. Finding a way to make GP synthesis more accessible while keeping it economically viable is not trivial.

### 1.2 Shortcomings of the state-of-the-art peptide synthesis processes

The standard method of SPPS relies on mixing the reactants by shaking (Merrifield, 1986). Shaking is a highly inefficient method of mixing, resulting in poor diffusion, particularly for porous particles such as those used in SPPS (Alshanski et al., 2018). To overcome this setback, several approaches were developed over the years. The most common protocol is employing a combination of a large excess of protected amino acids and reactants and a long reaction time to increase yields and avoid deletion sequences, using up to six equivalents of each AA and taking up to 2 hours per AA coupling (Wang et al., 2026). Use of this technique for the assembly of GPs is time-consuming, wasteful in precious protected GAAs and prone to unwanted side-reactions such as racemization or elimination (Barany et al., 1987). Additionally, this protocol is completely incompatible with streamlined post-assembly modifications which are required in GP synthesis.

The aforementioned impediments render the preparation of GP libraries hardly achievable, highlighting the need for an accelerated, atom-economical, streamlined, and user-friendly strategy for GP synthesis. State-of-the-art methods of peptide synthesis accelerate the process by various methods. Flow SPPS utilizes better mixing to that end, enabling assembly in cycles that take less than 1 min per AA (Gates and Hartrampf, 2020). However, it has a very poor atom economy, as each coupling requires a very large excess of protected AAs, which can vary from eight up to a thousand equivalents (Maki et al., 2025). Microwave (MW) SPPS accelerates the process through heating (Pedersen et al., 2010). An average peptide assembled by this technique requires about three equivalents of all protected AAs and takes several hours to complete on the solid support. While faster than standard SPPS, both Flow and MW SPPS are incompatible with post-assembly modifications.

**FIGURE 1:**
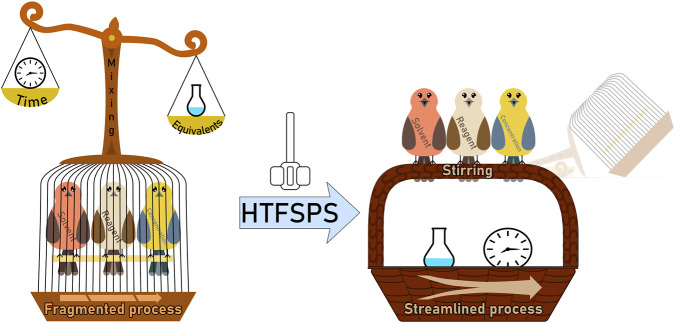
Left: In the state-of-the-art SPPS methods, inefficient mixing creates a clear tradeoff between reaction time and AA equivalents. The synthetic setups also create unavoidable constraints for solvent and reagent use and for reagent concentrations, and the synthesis of GPs must be done in a fragmented process. Right: By switching to HTFSPS protocols which employ high-shear stirring, it becomes possible to perform equimolar and fast reactions, and the setup allows process streamlining and great flexibility in reaction conditions.

There is a clear trade-off between shortening reaction times and increasing the equivalents of protected AAs, which exposes the limitations of current strategies for GP synthesis (Figure 1, left) (Isidro-Llobet et al., 2019). Moreover, contemporary protocols are typically rigid, enforcing fixed choices of solvent, reagents, and concentrations. Each approach therefore imposes a set of synthetic constraints that are difficult to circumvent. As a result, preparing even a single GP using the state-of-the-art methods is laborious, and extending these methods to synthesize GP libraries is, in practice, unfeasible.

### 1.3 The significance of diffusion for solid phase processes

A major key to reaction acceleration and optimization lies with understanding its kinetics. The kinetics of solid-phase reactions rely heavily on diffusion (Naoum et al., 2019). Unlike liquid-phase reactions, the diffusion in SPPS is not only dependent on mixing, but also on resin dispersion within the vessel and on an efficient permeation of solvents and reagents to and into the resin beads (Pinzón-López et al., 2023; 2025). Diffusion is dependent upon reagent concentration, temperature, reaction time, and mixing (Naoum et al., 2022). Even given other conditions, an effective mixing strategy is required for the achievement of fruitful interactions between the solvated protected and activated AAs and resin-anchored active sites.

**FIGURE 2:**
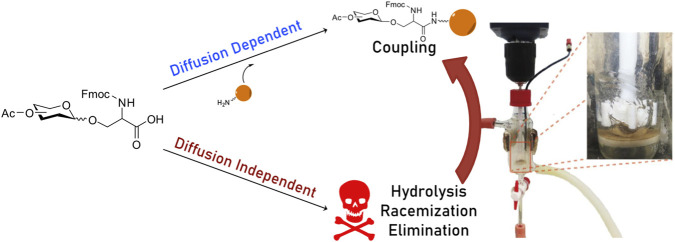
The two competing reaction pathways in SPPS. DD AA coupling competes with DI side-reactions that are destructive to the process. An efficient reaction should be conducted in conditions that promote DD over DI. Use of the HTFSPS reactor (right) allows these conditions to be obtained in fast and equimolar coupling reactions.

Reactions taking place in solid-phase synthesis can be divided generally into two categories by their kinetics: diffusion-dependent (DD) and diffusion-independent (DI). DD reactions (Figure 2, top), such as protected AA coupling or Fmoc deprotection, are characterized by reaction kinetics that are faster than the diffusion of solvents and reagents into the resin (Viklund et al., 1996; Wood and Gladden, 2002; Rudzinski and Plazinski, 2007). This makes diffusion the rate-limiting step in these reactions. In DD reactions, increasing the mixing efficiency will facilitate mass transfer via diffusion, and hence accelerate reactions. Conversely, DI reactions (Figure 2, bottom), such as the elimination or epimerization side reactions, are not affected by the rate of diffusion. Such reactions take place at similar rates regardless of mixing efficiency (Naoum et al., 2019). DI reactions do not benefit from improved mixing strategies. When using a large excess of reagents or conducting reactions for a long time, DD and DI reactions behave similarly. Only when trying to make the method as efficient as possible, the effects of diffusion dependence become pronounced.

Peptide assembly on solid support, and GP synthesis in particular, is governed by a continuous competition between desired DD reactions that form the target sequence and deleterious DI side reactions that generate by-products (Naoum et al., 2019). Under conditions of inefficient mixing and slow diffusion, DD and DI processes can proceed at comparable rates, favoring accumulation of side products. Because improved diffusion preferentially accelerates DD over DI pathways, it can be exploited to bias the system toward productive coupling and mitigate destructive side reactions. However, prevailing SPPS methodologies rely on mixing regimes that typically demand either high excess of protected AAs or prolonged reaction times to achieve sufficient diffusion. In our laboratory, we found that employing overhead mechanical stirring markedly enhances mixing efficiency (Alshanski et al., 2018).

### 1.4 Stirring without breaking: the principles of high-shear peptide synthesis

This discovery has led to further research aimed at expanding the practical applications of this technique (Naoum et al., 2019). Following process development, a reactor was prepared to enable the conduction of fast-stirring solid-phase reactions at elevated temperatures (Figure 2, right). A synthetic protocol compatible with the unique features of the reactor was established (Naoum et al., 2022). This protocol was named High-Temperature Fast-Stirring Peptide Synthesis (HTFSPS). The HTFSPS reactor is composed of a narrow glass tube with a sintered glass filter on one end and a double wall which can be used as a heating jacket. An overhead impeller is inserted from above through a hole in the cap to conduct the stirring, placed so that it does not touch the bottom or the walls. A feed insert is used for solution delivery and also acts as a baffle for vortex relapse. Testing of the reactor has shown that even after 7 hours of 1200 rpm stirring at 90 °C, resin beads remain intact (Naoum et al., 2022). Use of the simple HTFSPS reactor has allowed us to synthesize peptides at a good crude purity using equimolar quantities of protected AAs in 30 s per coupling, thus breaking the reaction time/AA equivalents tradeoff (Naoum et al., 2022). Moreover, this reactor allows high reproducibility of the synthetic protocol.

Following its initial development, HTFSPS was adapted and optimized to the Expeditious Glycopeptide Synthesis (EGPS) method, which enabled protected GAA coupling within 30 s while using only 1.2 equivalents of protected GAA, demonstrating a unique combination of speed and equimolarity in GP synthesis (Ben Abba Amiel and Hurevich, 2022). This method also includes a streamlined on-resin deacetylation stage that completes within 15 min. Deacetylation is commonly done in methanol and is thus incompatible with the state-of-the-art methods of GP synthesis (Lawrenson et al., 2017). The inclusion of a streamlined deacetylation step required the establishment of a new synthetic strategy, which was shown to be highly efficient. We then aimed to adjust EGPS for the synthesis of multiglycosylated peptides (Ben Abba Amiel et al., 2025). This demanded the adaptation of protocols to allow coupling reactions facing the much greater steric hindrance caused by multiple glycan moieties and also to deacetylate multiple sites simultaneously, both without forming significant amounts of deletion sequences and unwanted side-product. The adapted tactic involved an unorthodox use of activator excess, which allowed accessing GPs with multiple glycosylation sites and deacetylating them quickly and efficiently. The developed Expeditious Multiply Glycosylated Peptide Synthesis (EMGPS) strategy was further generalized by synthesizing GPs with a combination of O-Man and O-α-GalNAc cores (Ben Abba Amiel and Hurevich, 2026). The challenges associated with the synthesis were met by organizing steps to streamline the process, providing a new protocol termed Expeditious Heterogeneous Glycosylated Peptide Synthesis (EHGPS), which also entailed the development of an on-resin azide reduction step. The use of this setup has not only broken the reaction time/AA equivalents tradeoff, but also allowed us to overcome the other constraints that burden the state-of-the-art methods (Figure 1, right).

## 2 Diffusion-derived EGPS process evolution

As stirring promotes DD reactions and does not promote DI ones, they are performed in dissimilar reactors. The structure and function of the HTFSPS reactor make it ill-suited for the conduction of DI reactions, as it is much more useful for short, iterative reactions than for hours-long ones, while use of a shaker allows for long reactions to be conducted, often even several in parallel (Naoum et al., 2022). The interest of practicality demands the least number of transitions between the two types of setups. Therefore, each DI step requires the removal of the resin from the reactor and cannot be streamlined with DD steps. When we start to develop a module, such as deacetylation or azide reduction, we do not know whether it is DD, *i.e.*, whether it could be accelerated by diffusion-enhanced fast stirring. The answer to this question is detrimental to the entire process.

The difference between DD and DI reactions could facilitate steps that were hitherto considered inefficient or even impossible. An example for that could be seen in the coupling module which uses an excess of Hexafluorophosphate Azabenzotriazole Tetramethyl Uronium (HATU). It is commonly believed that use of an excess of HATU relative to the protected AA can result in an unwanted side reaction of guanidine formation (Yang, 2016). However, we have shown that the addition of excess HATU during coupling significantly improved conversion and did not form any unwanted side-products (Ben Abba Amiel et al., 2025). It is possible that given a long enough time, significant guanidine formation would take place, but that in our fast reaction strategy it is avoided strictly through the utilization of the fast kinetics of the coupling reaction.

Similarly, DI steps need to be planned so they do not interfere with the workflow. The reduction of azide to amide was introduced as a module during the latest adaptation effort of the protocol. We have attempted to conduct it expeditiously using fast stirring, but it invariably required a long reaction time (Ben Abba Amiel and Hurevich, 2026). Given that, we have reorganized the process so that it would not interfere with the DD coupling and deacetylation cycles. This allowed the development of a streamlined and more efficient strategy for synthesis of heterogeneously multiglycosylated peptides (HGPs). These differences have provided us with valuable observations which will be useful for future protocol development.

When conducting methodological studies, it is imperative to increase the level of difficulty gradually (Supplementary Image 1). Our first paper was focused on GPs bearing Xyl moieties on Ser (Ben Abba Amiel and Hurevich, 2022). Ser-Xyl is not only the least challenging GAA of the three to prepare, but also the easiest to incorporate in a GP. This enabled us to establish the fundamental conditions for the incorporation of simple protected GAAs into GPs, which could later be modified and adapted to more complex ones. The move to mannosylated and then to azidogalactosylated GAAs allowed a gradual increase in the synthetic difficulty, which could be dealt with based on the insights gained in previous stages. The work done on EGPS required many tests and optimizations (reaction conditions, racemization, elimination, etc.), which necessitated a protected GAA that could be easily prepared in large quantities. Drawing on insights from these experiments, we were able to begin assembling clustered MGPs for EMGPS (Ben Abba Amiel et al., 2025). This step, in turn, necessitated further optimization, which was facilitated by the convenient Ser-Man model. By the time we advanced to EHGPS, all individual modules had been sufficiently refined, and the only remaining task was the development of the reduction module (Ben Abba Amiel and Hurevich, 2026). Ultimately, the challenges associated with accelerated GP synthesis could be addressed only through a gradual, stepwise improvement of every component of the process.

## 3 The misconception of mixing and its contribution to underperforming SPPS processes

The scientific world is built on creating acceptable common grounds. Each scientific area has formed some unshakable assumptions and practices during the years. Accepted conceptions allow avoiding the tedious process of constantly challenging theories and practices. A researcher must rely on the knowledge gained by their predecessors in order to build new layers upon it. While working upon a preset assumption homogenizes science, accepting those blindly creates gaps which might be overcome only when conceptions are challenged and misconceptions are surfaced. As scientists, we must be vigilant that our conceptions are not misconceptions. Very often, we are not aware of the assumptions we make regarding our actions.

The notion that stirring cannot be used in solid-phase synthesis is regarded as an axiom, known since the strategy was first conceived by Merrifield in 1963 (Merrifield, 1963). However, a thorough examination of the initial papers presenting this method reveals that in fact there is no explicit mention of a need to avoid stirring (Merrifield, 1964a; 1964c; 1964b; 1965; Merrifield and Stewart, 1965; Najjar and Merrifield, 1966). Nonetheless, this conception is common knowledge among peptide chemists, so much so that it is taken for granted and not even mentioned in most works. This could be demonstrated by the fact that stirring is avoided in all commercial automated and manual SPPS technologies.

While it is true that stirring with a magnetic stirrer grinds the beads, other methods of stirring are similarly shunned (Alshanski et al., 2018; Bakhatan et al., 2020). This conception has cost the scientific community years of work with slow and inefficient setups, simply because researchers would not consider the possibility that a safe and easy way to stir exists. Generations of peptide chemists were told as young students that stirring is not to be done since the brittle solid support would certainly break. From that moment on, the idea was set in stone in their minds without a second thought. Old and new studies do not explain why fast stirring is not used for SPPS, just as they do not need to explain why it is used for other reactions.

It is well known that shaking is an inefficient mixing strategy. Over the years, many solutions were proposed to try to deal with this constraint, but without challenging its procedural origin. The state-of-the-art methods avoided the simple option of stirring, as it was believed such action would certainly grind the resin. The simple fact that magnetic stirring grinded resin types used in the 1960s meant that 60 years later the community still refrained from all kinds of fast stirring.

The high-shear mixing strategy was developed several years ago, and has since been optimized and tested on a large variety of products (Naoum et al., 2022; Bakhatan et al., 2023; Strauss et al., 2023). The fact that SPPS could be done under extremely fast mixing without breaking the beads was a surprise to the community, and to date it is still not easy to convince peptide chemists of the misconception they were taught in their training.

The observations we made could not have been obtained in other methods of SPPS. Only by breaking the stirring misconception could we arrive at a method in which diffusion dependence is so consequential, and only when diffusion dependence is consequential could we discern its effect on specific reactions.

All of these so-called “misconceptions” are not categorically untrue. They became axioms because they were observed under certain conditions, that may or may not apply for other settings. They were not challenged just for the sake of challenging, but for practical reasons. The question of when and how conceptions should be challenged does not have a clear answer. In our case, when the current strategy reached an impasse, then the interest of practicality dictated that a completely new approach should be tested. Just like module optimization or process streamlining, we view conception challenging as a practical tool. The surprise we are met with when we present this method to new audiences shows just how important this tool is and how deep can misconceptions be rooted.

## 4 A comparative study of GP SPPS methods

The synthesis of glycopeptides is not as common as that of standard peptides or even as that of peptides with simple modifications, e.g., phosphate, acetyl, etc. While the common SPPS strategy was reported already in the 1960s and GPs were prepared via a similar route for years, the ability to accelerate the process was only recently demonstrated. There is a limited number of cases in which an attempt to expedite the SPPS of O-GPs was carried out. MW-SPPS and Flow-SPPS are the state-of-the-art in peptide synthesis acceleration. EGPS was compared with the above strategies to highlight the differences between them. The comparison is not straightforward as the examples vary in methodology, sequence, length, glycosylation, and many other aspects. Some of these aspects are related to the specific technological differences, e.g., mixing method, which are interconnected with other parameters and make a precise comparison difficult. Nevertheless, we chose a number of representative recent examples that utilize Flow and MW for accelerated GP synthesis and determined the parameters that characterize the processes. While some parameters, e.g., reaction time and purified yield are easy to find, others, e.g., racemization and crude purity were not always reported. We used these parameters to compare with GP SPPS using EGPS (Supplementary Data Sheet 1).

Flow SPPS is an emerging technology to accelerate peptide synthesis. An exemplary report of flow SPPS of a GP highlights that the process is automated and fast, providing a medium size GP with a single glycosylation in decent yield and crude purity (Williams et al., 2024). However, the process is not streamlined in two aspects. First, the protected GAA coupling takes place via manual addition. Second, the deacetylation does not take place in flow. While this allows the introduction of the protected GAA with only two equivalents, there is no clear mention of racemization or what prevented the incorporation of the protected GAA as an integral part of the flow process (Supplementary Table S2, Entry [1]). Another report of flow SPPS of GPs incorporated the protected GAAs as part of the automated process, albeit at a very long coupling time, with each introduction of a protected GAA taking 2 h (Wang et al., 2026). This report is also not streamlined in that the deacetylation is done post-cleavage (Supplementary Table S2, Entry [2]).

Two exemplary MW-SPPS reports show that the preparation of medium and large peptides with up to twenty glycosylations (Supplementary Table S2, Entries [3] and [4]) (Galashov et al., 2024; 2025). The strategy combined MW-heating for standard AAs while the introduction of protected GAAs takes place at RT, in a different reaction time and in non-standard coupling solvents and conditions. The method is almost equimolar in GAAs, automated, and provides MGPs in a reasonable yield, but the crude purity and racemization are not quantitatively analyzed. Here as well, the deprotection is not streamlined with the process, which adds a post-assembly manipulation.

The EGPS process (Supplementary Table S2, Entry [5]) is entirely streamlined. The GAA coupling cycle time is very short, thus allowing a fast overall process (Ben Abba Amiel and Hurevich, 2022). Racemization studies with an analogous GAA isomer indicate only low racemization (below HPLC detectable range). The temperature was kept constant during the entire assembly and there was no need for condition changes. EMGPS and EHGPS (Supplementary Table S2, Entries [6] and [7]) maintained the same methodological guidelines, resulting in high crude purities after a very short assembly process (Ben Abba Amiel et al., 2025; Ben Abba Amiel and Hurevich, 2026). HPLC and NMR analysis of the resulting GPs indicated that the process does not lead to significant racemization even when multiple GAAs are introduced.

## 5 The future of EGPS as enabling tool for glycobiology

The work we have done so far can serve as the foundation for further developments. Glycosylations of proteins are very diverse. The type of the core, the specific extension and modification of the glycan combines with the multiple glycosylations sites, their heterogeneity and the co-existence with other PTMs to set a vast synthetic challenge. These hurdles have thus far limited the ability of synthetic constructs to mirror the diversity level associated with native glycoproteins, which prevents from elucidating the full scope of glycosylation significance. There is a great gap between the abundance and importance of multiple and heterogenous glycosylation of proteins and the ability to properly study them using synthetic GPs. This is even further complicated when longer glycan cores are concerned. Currently, there are limited ways to access GPs with extended glycans. The first one is by preparing GAA with the extended glycan prior to SPPS. The other option for glycan core elongation is chemoenzymatic, which is slow, limited to small quantities and unable to selectively perform asymmetric elongation (Ma et al., 2025). To date, both strategies are wasteful and lack the flexibility to prepare diverse sets of GPs. Preparation of large quantities of complex GAAs and the use of high-end SPPS machinery and processes makes the accessibility to complex GPs simply too expensive. While some approach this challenge computationally, a basic set of GPs is still required to develop and optimize the models (Wang et al., 2026). A robust method for the procurement of complex GPs with heterogeneous and longer glycan cores is desperately needed.

Our work on MGPs and HGPs opens a path that may lead to the procurement of even more complex GPs. The flexibility and simplicity of the HTFSPS derived processes and the use of an accessible, user-friendly and cheap-to-build setup will be fundamental in complex GP synthesis. There are several possible routes by which the essence of EGPS can become useful. The ability to execute various post-assembly deprotection and modification steps in a streamlined manner could allow obtaining HGPs with orthogonally-protected glycan moieties, which would then allow asymmetric chemoenzymatic or chemical elongation, greatly increasing the scope of available GPs. Fast-stirring SPPS is not limited to O-GPs and was demonstrated for the fast synthesis of phosphopeptides and N-linked glycopeptides (Grunhaus et al., 2022; Strauss et al., 2023). The accessibility to GPs with complex and large glycans, preferably in multiple positions, via EMPGS strategies can address the gap between their importance in biological systems and the limited accessibility to these valuable compounds. The most challenging aspect enroute to PGs with extended and complex glycan cores still lies in the scarce availability of the GAAs. This is added to the difficulty in introducing such huge and complex moieties to the peptides via SPPS, which might further increase coupling deficiencies, aggregation propensity, and solubility-related problems. The development of effective methods that maximize DD reactions could allow the coupling of GAAs with longer glycan cores, thereby even further expanding the potential of the process. As we advance the chemical and the engineering aspect of EGPS, developing ways to synthesize peptides that combine several kinds of PTMs will also be attractive.

The demonstrated utility of generalized EGPS could be leveraged for a vast variety of enterprises. The easy adaptability and optimization of each step of the process lends itself to further generalization. The high throughput supported by this modular protocol can benefit both upscaling and the preparation of libraries. The versatility of EGPS processes may serve as an invaluable tool for glycobiology research and be applicable for novel GP-derived applications, as demonstrated for bacterial biosensing (Ben Abba Amiel et al., 2025). On top of this, the intrinsic simplicity and practicality of this technique could facilitate its widespread adoption in various settings as a vital tool for chemists and biologists alike.

## Data Availability

The original contributions presented in the study are included in the article/Supplementary Material, further inquiries can be directed to the corresponding author.

## Supplementary material

SUPPLEMENTARY TABLE S1 A table describing the evolution of each synthetic module in the transitions between methods. The work started with the condition defined for HTFSPS (left), and then modules were added and modified in each generalization of the method according to need. When the last stage of the process, i.e., EHGPS (right), was reached, the only change to the process that was required was the addition of an azide reduction step, indicating that previous stages were adapted efficiently. All modules were stirred at 1200 rpm, except for azide reduction and cleavage, which were done by shaking. All modules were done at 90 °C in DMF unless otherwise stated.

SUPPLEMENTARY TABLE S2 A comparative table that describes the key parameters for accelerated SPPS of GPs and highlights strategy-related differences.
