# Supplementary figures and images for: Stirring glycopeptides away from the constraints of solid-phase synthesis misconceptions
Source: Front Mol Biosci. 2026 Jun 10;13:1863326. doi: 10.3389/fmolb.2026.1863326 (PMC13290445; doi:10.3389/fmolb.2026.1863326)

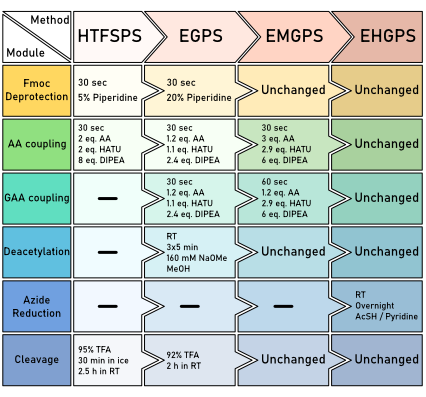

Supplement: Supplementary file 1 [file Image1.tiff]
