# Supplementary material for: Stirring glycopeptides away from the constraints of solid-phase synthesis misconceptions
Source: Front Mol Biosci. 2026 Jun 10;13:1863326. doi: 10.3389/fmolb.2026.1863326 (PMC13290445; doi:10.3389/fmolb.2026.1863326)
Supplement: Supplementary file 2 [file DataSheet1.pdf]

Reference*	Method	GAA Equiv.	Assembly temperature (⁰C)<br>AA<br>GAA	Sequence length (Max Units)	Glycosylations:<br>Max No.<br>Type	Total time of synthesis<br>GAA coupling time	Yield	Crude Purity	Racemization	Mixing<br>AA<br>GAA	Deacetylation:<br>Post assembly<br>Streamlined	Manual / Automated
[1]	Flow + Manual GAA coupling	2	Varied<br>90<br>RT	14 AA	X 1<br>Thr-β-GlcNAc	>150min<br>90 min	32%	71%	-	Flow<br>Shaking	N_2H_4, 30 min x2<br>On-Resin	Auto + Man
[2]	Flow	2	Constant<br>RT<br>RT	27 AA	X 9<br>Ser\Thr α-Man\α-Gal\α-Glc	>10h<br>120 Min	6%-18%	-	-	Flow	N_2H_4, 30 min<br>After cleavage	Auto
[3]	MW	1.5	Varied<br>90<br>RT	40 AA	X 10<br>Ser\Thr-α-GalNAc	>17h<br>10 min	9%-17%	-	-	Oscillation	N_2H_4, 30 min<br>On-Resin	Auto
[4]	MW	1.5	Varied<br>90<br>RT	41 AA	X 20<br>Ser\Thr-α-GalNAc	>4h<br>10 min	2%-27%	-	-	Oscillation	N_2H_4, 30 min<br>On-Resin	Auto
[5]	EGPS	1.2	Constant<br>90<br>90	9 AA	X 1<br>Ser-β-Xyl	<2h<br>30 sec	-	55%-83%	None	Fast overhead stirring	NaOMe, 15 min<br>On-resin	Man
[6]	EMGPS	1.2	Constant<br>90<br>90	13 AA	X 3<br>Ser\Thr-α-Man	<2h<br>1 min	11%-13%	~70-80%	None	Fast overhead stirring	NaOMe, 15 min<br>On-resin	Man
[7]	EHGPS	1.2	Constant<br>90<br>90	9 AA	X 2<br>Ser\Thr-α-Man<br>Ser\Thr-α-GalNAc	<2h<br>1 min	22%-62%	~50-85%	None	Fast overhead stirring	NaOMe, 15 min<br>On-resin	Man

GAA: Glycosylated Amino Acid
MW : microwave
EGPS: Expeditious Glycopeptide Synthesis
EMGPS: Expeditious Multiglycopeptide Synthesis (multiple glycans)
EHGPS: Expeditious Heteroglycopeptide Synthesis (multiple and different glycan cores on the same peptide)
*See references in the next page

References:

[1] Williams, E. T., Schiefelbein, K., Schuster, M., Ahmed, I. M. M., De Vries, M., Beveridge, R., et al. (2024). Rapid flow-based synthesis of post-translationally modified peptides and proteins: a case study on MYC's transactivation domain. *Chem. Sci.* 15, 8756–8765. doi: 10.1039/D4SC00481G

[2] Wang, R., Liu, W., Li, X., Zhang, Y., Tian, J., Zhu, H., et al. (2026). Deciphering How Clustered O-Glycosylation Shapes Substrate-Binding Preferences in an Intrinsically Disordered Protein Region. *JACS Au* 6, 1977–1987. doi: 10.1021/jacsau.6c00079

[3] Galashov, A., Kazakova, E., Stieger, C. E., Hackenberger, C. P. R., and Seitz, O. (2024). Rapid building block-economic synthesis of long, multi-O-GalNAcylated MUC5AC tandem repeat peptides. *Chem. Sci.* 15, 1297–1305. doi: 10.1039/D3SC05006H

[4] Galashov, A., di Gregorio, E., Ponomareva, P., Safferthal, M., Kazakova, E., Bechtella, L., et al. (2025). Impact of Glycoclustering on Stiffening of MUC5AC Peptides Revealed by High-Efficiency Synthesis. *Angew. Chem. Int. Ed.* 64, e202508278. doi: 10.1002/anie.202508278

[5] Ben Abba Amiel, D., and Hurevich, M. (2022). Expeditious Synthesis of a Glycopeptide Library. *European J. Org. Chem.* 2022. doi: 10.1002/ejoc.202200623

[6] Ben Abba Amiel, D., Okshtein, H., Alshanski, I., Hayouka, Z., Yitzchaik, S., and Hurevich, M. (2025). Expeditious Synthesis of Multiglycosylated Peptides for Sensing of Listeria monocytogenes. *J. Med. Chem.* 68, 26513–26524. doi: 10.1021/acs.jmedchem.5c02818

[7] Ben Abba Amiel, D., and Hurevich, M. (2026). Expeditious synthesis of multiglycopeptides with heterogeneous glycan cores derived from an α-dystroglycan mucin-like domain. *Org. Biomol. Chem.* 24, 1889–1898. doi: 10.1039/D6OB00013D
